# Population Genomic Analysis of 1,777 Extended-Spectrum Beta-Lactamase-Producing *Klebsiella pneumoniae* Isolates, Houston, Texas: Unexpected Abundance of Clonal Group 307

**DOI:** 10.1128/mBio.00489-17

**Published:** 2017-05-16

**Authors:** S. Wesley Long, Randall J. Olsen, Todd N. Eagar, Stephen B. Beres, Picheng Zhao, James J. Davis, Thomas Brettin, Fangfang Xia, James M. Musser

**Affiliations:** a Center for Molecular and Translational Human Infectious Diseases Research, Department of Pathology and Genomic Medicine, Houston Methodist Research Institute and Houston Methodist Hospital, Houston, Texas, USA; b Department of Pathology and Laboratory Medicine, Weill Cornell Medical College, New York, New York, USA; c Computing, Environment and Life Sciences, Argonne National Laboratory, Argonne, Illinois, USA; d Computation Institute, University of Chicago, Chicago, Illinois, USA; MedImmune

## Abstract

*Klebsiella pneumoniae* is a major human pathogen responsible for high morbidity and mortality rates. The emergence and spread of strains resistant to multiple antimicrobial agents and documented large nosocomial outbreaks are especially concerning. To develop new therapeutic strategies for *K. pneumoniae*, it is imperative to understand the population genomic structure of strains causing human infections. To address this knowledge gap, we sequenced the genomes of 1,777 extended-spectrum beta-lactamase-producing *K. pneumoniae* strains cultured from patients in the 2,000-bed Houston Methodist Hospital system between September 2011 and May 2015, representing a comprehensive, population-based strain sample. Strains of largely uncharacterized clonal group 307 (CG307) caused more infections than those of well-studied epidemic CG258. Strains varied markedly in gene content and had an extensive array of small and very large plasmids, often containing antimicrobial resistance genes. Some patients with multiple strains cultured over time were infected with genetically distinct clones. We identified 15 strains expressing the New Delhi metallo-beta-lactamase 1 (NDM-1) enzyme that confers broad resistance to nearly all beta-lactam antibiotics. Transcriptome sequencing analysis of 10 phylogenetically diverse strains showed that the global transcriptome of each strain was unique and highly variable. Experimental mouse infection provided new information about immunological parameters of host-pathogen interaction. We exploited the large data set to develop whole-genome sequence-based classifiers that accurately predict clinical antimicrobial resistance for 12 of the 16 antibiotics tested. We conclude that analysis of large, comprehensive, population-based strain samples can assist understanding of the molecular diversity of these organisms and contribute to enhanced translational research.

## IMPORTANCE

*Klebsiella pneumoniae* causes human infections that are increasingly difficult to treat because many strains are resistant to multiple antibiotics. Clonal group 258 (CG258) organisms have caused outbreaks in health care settings worldwide. Using a comprehensive population-based sample of extended-spectrum beta-lactamase (ESBL)-producing *K. pneumoniae* strains, we show that a relatively uncommon clonal type, CG307, caused the plurality of ESBL-producing *K. pneumoniae* infections in our patients. We discovered that CG307 strains have been abundant in Houston for many years. As assessed by experimental mouse infection, CG307 strains were as virulent as pandemic CG258 strains. Our results may portend the emergence of an especially successful clonal group of antibiotic-resistant *K. pneumoniae*.

## INTRODUCTION

*Klebsiella pneumoniae* is a major human pathogen responsible for high morbidity and mortality rates (1–3). Numerous studies have investigated the genetic epidemiology of *K. pneumoniae* infections in hospitalized patients in the United States and elsewhere (3–8). These studies have often used low-resolution molecular typing such as multilocus sequence typing (MLST) or have studied relatively small collections of organisms. Studies of hospitalized patients in the United States commonly have reported a predominance of clonal group 258 (CG258) strains (9–12). CG258 was first identified in 2009 as a single-locus variant of sequence type 11 (ST11) from Hungary (13) and has since been reported worldwide. Data suggest that CG258 diverged from its most recent common ancestor in 1995, after acquisition of the *K. pneumoniae* carbapenemase (KPC) gene (14).

*K. pneumoniae* strains producing KPC carbapenemase were first identified in North Carolina in 1996 (15) and now cause multidrug-resistant hospital-acquired infections globally (16). Evidence suggests that there is circulation of *K. pneumoniae* clones within hospitals, with transmissible resistance plasmids shared between *Klebsiella* spp*.* and other pathogens (5, 17–20). This sharing of resistance elements is believed to have contributed to the emergence of multidrug resistance in *K. pneumoniae* and large, hospital-based outbreaks. The increasing reports of these nosocomial outbreaks are of great clinical concern (21, 22).

To develop new therapeutics and control strategies for *K. pneumoniae*, it is imperative to understand the population genomic structure of strains causing human infections. To begin filling this knowledge gap, we sequenced the genomes of 1,777 extended-spectrum beta-lactamase (ESBL)-producing *K. pneumoniae* strains (Table 1; see Table S1A to C in the supplemental material) cultured from patients in the 2,000-bed Houston Methodist Hospital system between September 2011 and May 2015. These strains were all positive for ESBL production, which accounted for an average of 40% of the *K. pneumoniae* strains in our laboratory during the study period. The Houston Methodist Hospital system serves patients in the greater Houston metropolitan area (population of approximately 6,000,000), now the most ethnically diverse population center in the United States (23). In addition to our large and diverse local population, patients routinely travel for treatment to Houston Methodist Hospital from across the United States and internationally. The genome sequence data identified unexpectedly high diversity. Strains varied markedly in gene content and had an extensively diverse assortment of large plasmids. These transmissible plasmids often contain antimicrobial resistance genes and represent important elements contributing to the dissemination of resistance markers.

10.1128/mBio.00489-17.4 TABLE S1  (A) Summary of the CGs/STs, capsule loci, collection dates, sources, yersiniabactin loci, and colibactin loci of 1,777 *K. pneumoniae* strains. (B) Summary of selected antimicrobial resistance genes. (C) Summary of the top six plasmid replicons from 1,777 *K. pneumoniae* strains. Download TABLE S1, XLSX file, 0.3 MB. Copyright © 2017 Long et al. 2017 Long et al. This content is distributed under the terms of the Creative Commons Attribution 4.0 International license.

**TABLE 1:** Summary of clonal groups and specimen sources of 1,777 *K. pneumoniae* isolates

Source(s)	No. of isolates (% of total)			
	CG258	CG307	Other CG	Total
Blood	34 (1.9)	54 (3.0)	59 (3.3)	147 (8.3)
Respiratory	130 (7.3)	111 (6.2)	143 (8.0)	384 (21.6)
Urine	220 (12.4)	374 (21.0)	333 (18.7)	927 (52.2)
Other^a^	90 (5.1)	105 (5.9)	124 (7.0)	319 (18.0)
All	474 (26.7)	644 (36.2)	659 (37.1)	1,777 (100)

^a^ Other specimen sources include tissue, wounds, and body fluids.

## RESULTS

### Whole-genome sequencing of 1,777 ESBL-producing *K. pneumoniae* strains identified extensive genomic diversity within and between major clades.

**FIG 1:**
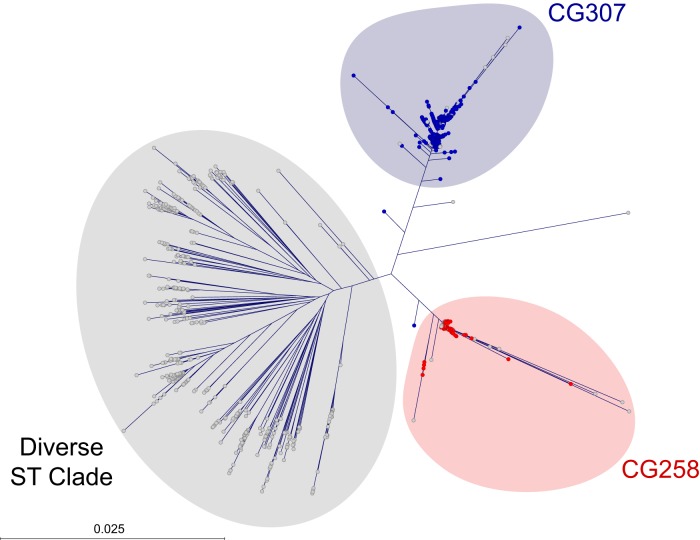
Estimates of genetic relationships among 1,777 ESBL-producing *K. pneumoniae* strains recovered from infected patients in a multiple-hospital system in the Houston metropolitan area. Polymorphisms were called against the genome of CG258 *K. pneumoniae* reference strain NJST258_2 (NCBI GenBank accession no. CP006918.1). Colors represent the major clonal groups, including CG258 (red) and CG307 (blue), and the remaining heterogeneous STs (gray). Phylogenetic relationships were defined by the neighbor-joining method in FastTreeMP with double precision. The core genome was defined as the chromosomal sequence with phage sequence regions excluded. To simplify presentation, outliers have been cropped out of frame at 3 and 9 o’clock. The outlier strains, which were identified as *K. pneumoniae* by matrix-assisted laser desorption ionization mass spectrometry and MLST, may be allied with the genomes of some strains referred to as *K. quasipneumoniae* and *K. variicola*.

We sequenced the genome of 1,777 ESBL-producing *K. pneumoniae* strains (Table 1 and Fig. 1; see Table S1A to C and Fig. S1) cultured between September 2011 and May 2015 from infected patients in the 2,000-bed Houston Methodist Hospital system. The sample represents virtually all of the ESBL-producing *K. pneumoniae* strains recovered during this time period. Over half of the strains were cultured from urine (927 strains, 52.2%). The remainder were cultured from respiratory (384 strains, 21.6%), blood (147 strains, 8.3%), and other tissue, wound, and miscellaneous specimens (319 strains, 18.0%).

10.1128/mBio.00489-17.1 FIG S1  Antimicrobial agent resistances and plasmid contents of ESBL-producing *K. pneumoniae* strain*s*. Plasmid replicons and antimicrobial resistance gene contents were identified by using SRST2. Neighbor-joining phylogenetic relationships relative to NJST258_2 are shown in a cladogram for the major clades CG258 (red) and CG307 (blue) and the remaining heterogeneous diverse CGs (gray). The presence of a KPC gene allele is indicated in the first track as KPC-2 (green) or KPC-3 (purple). The six tracks to the right indicate the presence (black) or absence (white) of plasmid replicons in the replicon types listed. To prevent bias from multiple strains collected from the same patient, only the first isolate per patient is shown. Download FIG S1, PDF file, 0.4 MB. Copyright © 2017 Long et al. 2017 Long et al. This content is distributed under the terms of the Creative Commons Attribution 4.0 International license.

**FIG 2:**
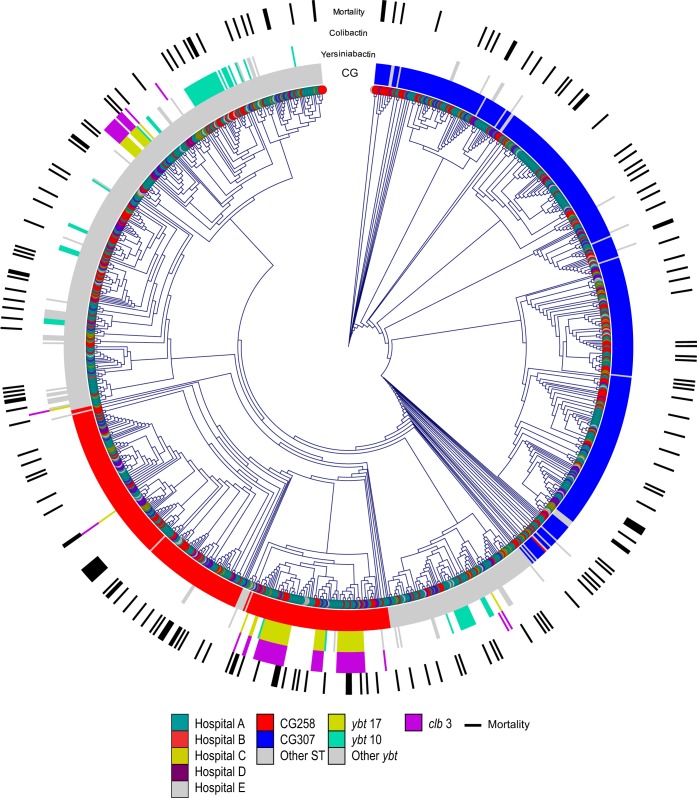
Spatial relationships of ESBL-producing *K. pneumoniae* strains from the multiple hospitals in the Houston Methodist system with clonal groups and associated patient deaths. This circular cladogram displays the first isolate from each patient, color coded by the five hospitals of origin (teal, yellow, red, purple, and gray). The circles surrounding the cladogram, from innermost to outermost, indicate the clonal group of the strain, yersiniabactin locus, colibactin locus, and patient death. In the innermost circle, the CG258 strains are red and the CG307 strains are blue. Other clonal types are indicated with a gray bar. The yersiniabactin circle is color coded to indicate which yersiniabactin locus is present, if any, with the two most common loci, *ybt17* and *ybt10*, in yellow and teal, respectively; all other *ybt* loci are gray. The colibactin circle is purple to indicate the presence of *clb3*, the only colibactin synthesis locus detected in our collection. In the outermost circle, in-hospital death is indicated by a black line. For patients with multiple strains, only the first isolate from the patient is represented on the tree.

**FIG 3:**
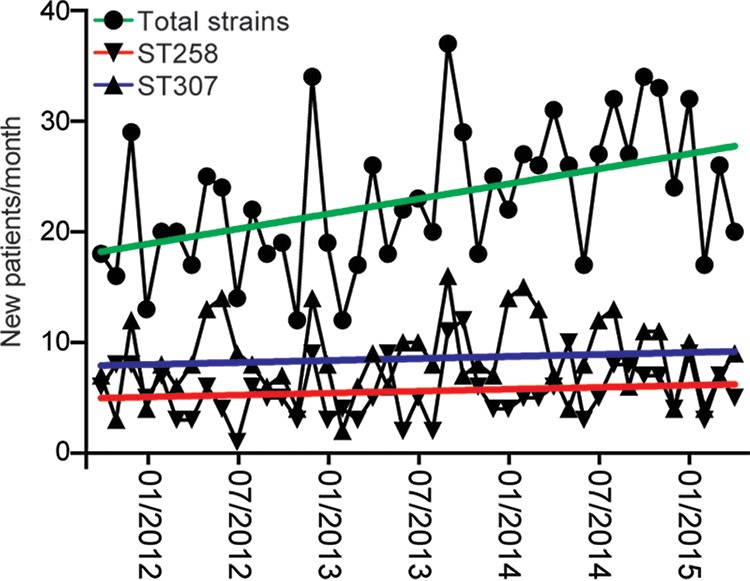
ESBL-producing *K. pneumoniae* strains recovered during the course of this study. The first strain recovered from each patient is graphed (circles, green linear regression). The number of strains classified as CG307 (triangles, blue linear regression) and CG258 (inverted triangles, red linear regression) are also shown. This figure demonstrates that CG258 and CG307 strains were abundant throughout the study period.

Much of the literature describing nosocomial outbreaks or hospital strain samples in the United States and elsewhere has reported a predominance of CG258 strains, regardless of the population studied (17, 24–29). In striking contrast, on the basis of genome-wide phylogenetic analysis, our strains were broadly classified into three major genetic clades (Fig. 1; Table 1). Approximately one-fourth (474 strains, 26.7%) were assigned to CG258. These organisms represent progeny of a globally disseminated epidemic clone reported to be the major cause of *K. pneumoniae* infections in the United States (1, 13, 14, 30–32). The average minimum pairwise genetic distance (a measure of genomic diversity in a population of related strains) between the CG258 strains in our sample was 346 single nucleotide polymorphisms (SNPs). More than one-third of the strains (635, 35.7%) were assigned to CG307. These organisms were cultured from patients at all of the hospitals in our system; that is, no single hospital was responsible for the abundance of CG307 infections (Fig. 2). Similarly, CG258 strains caused infections in patients at all of our hospitals (Fig. 2). CG307 and CG258 strains were common in our population throughout the study period (Fig. 3).

Although strains of CG307 have been identified sporadically in diverse locations globally in recent years (10, 33–40), they have not been reported to be abundant causes of infections in a large unbiased population over a multiyear period. The average minimum pairwise genetic distance between the CG307 strains was 948 SNPs. Compared to CG258 organisms that have been extensively studied, very little is known about strains of CG307. The remaining strains (668 strains, 37.6%) in our sample were a genetically heterogeneous array of multilocus STs reported to cause infections worldwide. The average minimum pairwise genetic distance among these 668 strains is 6,580 SNPs, reflecting the far greater diversity present in these strains than in CG258 and CG307 organisms. There were 99 unique previously defined STs present in the strains sequenced (1,430 strains, 80.4%) (see Table S2). An additional 51 strains (2.9%) had novel combinations of known ST alleles, whereas the remaining 296 strains (16.7%) have novel ST alleles resulting in previously undefined ST types. After CG258 and CG307, the three more common STs were ST16 (92 strains, 5.2%), ST15 (49 strains, 2.8%), and ST280 (32 strains, 1.8%).

10.1128/mBio.00489-17.5 TABLE S2  STs of the strains recovered in this study. Download TABLE S2, PDF file, 0.1 MB. Copyright © 2017 Long et al. 2017 Long et al. This content is distributed under the terms of the Creative Commons Attribution 4.0 International license.

### Closure and annotation of five new *K. pneumoniae* reference genomes.

Five strains were sequenced to closure using PacBio long-read sequencing (see Table S3). This allowed the annotation of new reference strains to aid our studies of *K. pneumoniae* population genomics and pathogenesis. The five strains were chosen to represent regions of the phylogenetic tree for which existing reference genomes deposited in publically available databases were lacking. In addition, genomes containing the *bla*_NDM-1_ and OXA-48 genes (see below) were chosen to allow more in-depth analysis of these important strains.

10.1128/mBio.00489-17.6 TABLE S3  Strains sequenced to closure in this study. Download TABLE S3, PDF file, 0.04 MB. Copyright © 2017 Long et al. 2017 Long et al. This content is distributed under the terms of the Creative Commons Attribution 4.0 International license.

KPN11 is representative of the CG307 strains in our patient population. KPN528 was chosen for PacBio sequencing and closure because it was the first *K. pneumoniae* strain in our sample identified with the *bla*_NDM-1_ metallo-beta-lactamase. KPN1481 (ST906) was chosen because it was one of the last strains in our sample containing the *bla*_NDM-1_ metallo-beta-lactamase gene, and a closed reference genome has not been reported for this clonal type. Similarly, KPN1482 (ST37) is a clonal type for which a closed reference genome is not available. BK13043, a representative CG258 strain collected in New York City in 2004, was chosen to provide a historical CG258 reference genome.

Each new closed genome contained two to five plasmids (see Table S3). The plasmids matched previously identified reference plasmids in GenBank with sequence coverage ranging from 21 to 100%. The *bla*_NDM-1_ containing plasmids present in KPN528 and KPN1481 differed. KPN528 had a 292-kb plasmid that had 99% coverage of the pPKPN1 plasmid of reference strain PittNDM01, whereas strain KPN1481 had a 342-kb plasmid that had 74% coverage of the pNDM-MAR plasmid (41, 42). Strain KPN528 contained a second plasmid (76 kb) with 99% coverage of plasmid 3 present in strain PittNDM01. The PittNDM01 *K. pneumoniae* strain is an ST14 organism isolated from a patient in Pittsburgh, PA, in 2013 (41). Of note, KPN528 also is an ST14 strain and was isolated in December 2012. Taken together, these results suggest that these Pittsburgh and Houston strains are lineally descended from a common ancestor organism.

### Strains of CG307 are unexpectedly abundant.

KPC-producing *K. pneumoniae* were initially described in the Houston region in 2009 (43). That initial report was followed by the identification of multiple clonal types in 2010, including CG258 and CG307 (10). The CG307 clade of *K. pneumoniae* was first identified in a Dutch teaching hospital in 2008 (44). Since then, CG307 strains have been identified periodically in Europe, Africa, the Middle East, Asia, Central and South America, and New York City (20, 33–40, 45–47), and a hospital-based outbreak was recently reported in South Korea (48). However, they have not been documented to be of great abundance in any of these areas. Thus, in our genomic data set based on comprehensive, population-based sampling of ESBL-producing *K. pneumoniae* strains, we demonstrate the existence of a numerically highly successful but poorly characterized genetic group (CG307) with disease prevalence exceeding that of the well-known epidemic CG258 organisms. We identified no statistically significant difference between the observed in-hospital mortality rate of all patients infected with CG307 strains and that of patients infected with the CG258 clonal type (chi-square test) (Fig. 2). Similarly, the mortality rate did not differ statistically significantly between patients with bloodstream infections caused by CG307 and CG258 strains (chi-square test). Furthermore, no significant differences between CG307 and CG258 strains were identified in any clinical feature (see Table S1A), including the hospital of origin, organ transplant status, infection type (urinary tract versus invasive infection), and number of episodes of care (single versus multiple strains recovered longitudinally from the same patient).

### Comparison of CG258 and CG307 genomes: abundant variation in gene content.

**FIG 4:**
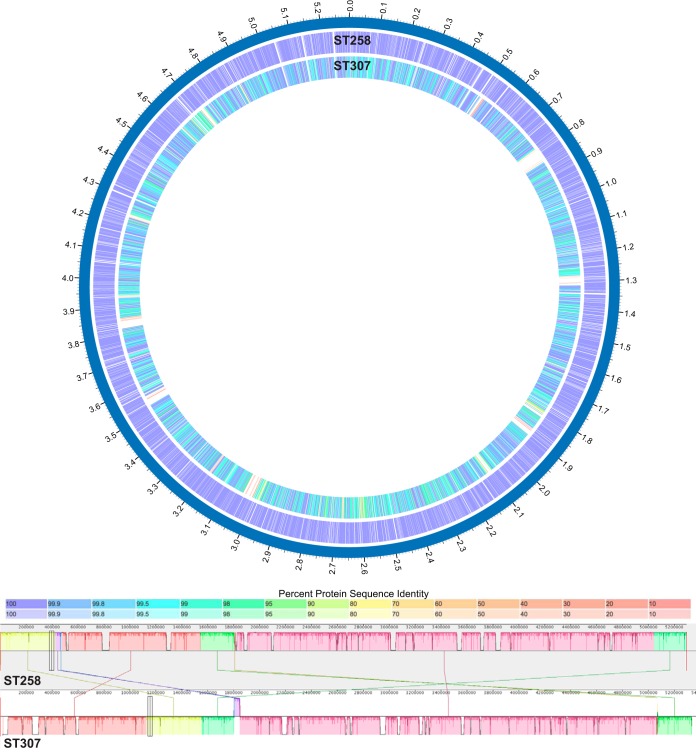
Gene content differences between the reference genomes of CG258 and CG307. CG258 is the globally abundant strain of *K. pneumoniae*, and CG307 is the locally abundant strain in our study. Bidirectional BLAST was performed by using the PATRIC resource to illustrate the differences in gene content between these two reference genomes. The color indicates the percent identity of the BLAST hit for each gene, with darker shading indicating a bidirectional hit and lighter shading indicating a unidirectional hit (top). A progressive Mauve alignment of the CG258 and CG307 reference genomes is shown. Local colinear blocks are organized by color (bottom). NJST258_2 is the CG258 reference genome, and KPN11 is the CG307 reference assembly.

The CG258 reference genome (NJST258_2; NCBI GenBank accession no. CP006918.1) has 5,188 annotated genes. Comparison of these genes to our assembled CG307 reference genome (KPN11) by bidirectional BLAST analysis found that 429 of the annotated NJST258_2 genes do not have a significant homolog present in the KPN11 CG307 genome (Fig. 4). Most of these genes (253 out of 429) are annotated as hypothetical proteins. Many of the others are associated with phage, secretion systems, or metabolism. Of the 5,237 annotated genes in the KPN11 CG307 reference assembly, there are 414 annotated genes that lack a homolog in NJST258_2, most of which (281 of 414) are annotated as hypothetical proteins. Most of the other genes that are missing are associated with phage, transduction elements, or metabolism. Some of the genes present in or absent from either clonal type are related to the capsule gene locus. Genetic variation in this locus is well established (17, 49, 50). A summary of the annotated genes present in or absent from NJST258_2 compared to KPN11 CG307 is presented in Tables S4 and S5. Variations in yersiniabactin locus, antimicrobial resistance gene content, and capsule locus are discussed in more detail below.

10.1128/mBio.00489-17.7 TABLE S4  Annotated genes present in NJST258_2 but not in CG307 strain KPN11. Download TABLE S4, PDF file, 0.1 MB. Copyright © 2017 Long et al. 2017 Long et al. This content is distributed under the terms of the Creative Commons Attribution 4.0 International license.

10.1128/mBio.00489-17.8 TABLE S5  Annotated genes present in KPN11 but not in NJST258_2. Download TABLE S5, PDF file, 0.1 MB. Copyright © 2017 Long et al. 2017 Long et al. This content is distributed under the terms of the Creative Commons Attribution 4.0 International license.

### Presence of yersiniabactin and colibactin loci in ESBL-producing *K. pneumoniae*.

Recent studies have emphasized the important contribution of siderophore gene acquisition to severe *Klebsiella* invasive disease (51, 52). Holt et al. reported that the yersiniabactin locus (*ybt*) was present in 40.0% of the CG258 and 32.2% of the overall *K. pneumoniae* genomes analyzed (52). In contrast, we found that only 274 of our 1,777 strains (15.4%) had the *ybt* locus. Only 13 of the 644 CG307 strains (2.0%) and 85 of the 474 CG258 strains (17.9%) contained the *ybt* locus. Of the 474 CG258 strains, 76 (16.0%) had both the colibactin synthesis locus (*clb*) and the *ybt* locus. In contrast, none of the CG307 strains carried a *clb* locus. The presence or absence of the yersiniabactin and colibactin loci is shown in Fig. 2 and presented in Table S1A. In contrast to results from other studies using smaller strain collections (51, 52), the presence of the yersiniabactin and colibactin loci was not significantly associated with any clinical feature, including invasive disease, chronic infection, or death (no statistically significant difference, chi-square test).

### Extensive plasmid diversity within ESBL-producing *K. pneumoniae*.

*K. pneumoniae* strains commonly have large plasmids that often contain antibiotic resistance genes and multiple replicons. The genome data identified extensive plasmid diversity among the 1,777 strains. The average number of plasmid replicons per isolate was 4.5 (standard deviation, 2.5; range, 0 to 16). Six replicons were each present in more than 25% of the 1,777 strains, i.e., FIBk (1,327 strains), FIIk (1,237 strains), FII (693 strains), R (667 strains), ColRNAI (660 strains), and FIBpQil (494 strains).

The distribution of the replicons across the phylogenetic tree is shown in Fig. S1 and Table S1C. The FIBk and FIIk replicons are present in strains of diverse clonal backgrounds, with some clustering in CG307 organisms. The FII, ColRNAI, and FIBpQiL replicons appear to cluster predominantly with CC258 strains, whereas the R replicons are associated with the CG307 organisms. The most common combination of replicons in a single isolate was FIBk, FII, ColRNAI, and FIBpQil (248 strains), followed closely by FII and FIBpQil (244 strains) and FIBk and FIBpQiL (228 strains). The only replicon of the top six to be found in the absence of any of the other five was FIBpQiL (177 strains).

### Diversity of ESBL genes.

Our analysis identified an extensive array of ESBL genes in the 1,777 genomes, including those encoding carbapenemases. Genes in the SHV-LEN-OKP family of ESBLs were most common, being present in 1,442 of the 1,777 strains. The second most common ESBL gene was *bla*_CTX-M_, which was present in 1,161 of 1,777 strains (see Table S1B). Among the eight *bla*_CTX-M_ alleles identified, *bla*_CTX-M-15_, was the most abundant. Other ESBL-encoding genes identified included a member of the TEM family (797 strains) and the KPC gene (581 strains). OXA genes characterized as encoding ESBLs (OXA-23, OXA-24, OXA-48, and OXA-83) were far less common, being present in only 23 strains. *bla*_NDM-1_-positive strains were cultured from six patients, two of whom had strains that also had the OXA-48 gene. Some of the antimicrobial resistance genes detected in these strains, such as TEM30, TEM33, and SHV26, are known to be inhibitor resistant.

Overall, KPC alleles were present in 64.6% of the CG258 strains (306 strains) compared to 34.3% of the CG307 strains (221 strains). Among all of our strains with a *bla*_KPC_ carbapenemase gene (581 strains), *bla*_KPC-2_ was the dominant allele (572 strains, 98.4%), including 293 CG258 strains (96.4%) and 197 CG307 strains (93.4%). Our results differ from those of other studies reporting that both *bla*_KPC-2_ and *bla*_KPC-3_ are found in CG258 *K. pneumoniae* (10, 13, 17, 20, 45, 53) and that CG307 was strongly associated with *bla*_KPC-3_ (39). CG307 strains expressing *bla*_KPC-2_ have been recently reported in Korea and Colombia (34–36). In our sample, no CG258 strains and only three CG307 strains had the *bla*_KPC-3_ allele. The difference between *bla*_KPC-2_ and *bla*_KPC-3_ is one nonsynonymous SNP (C814T) that results in an H272Y amino acid replacement. Whereas both *bla*_KPC-2_ and *bla*_KPC-3_ are very efficient at hydrolyzing penicillins, cephalosporins, and carbapenems, *bla*_KPC-3_ has increased enzymatic activity against and confers higher resistance to cephamycins and ceftazidime (15).

Four newer KPC variants (*bla*_KPC-7_, *bla*_KPC-8_, *bla*_KPC-9_, and *bla*_KPC-10_) likely derived from *bla*_KPC-3_ have a second nonsynonymous SNP that confers greater catalytic activity against ceftazidime than *bla*_KPC-2_ (54). That is, the use of ceftazidime worldwide may be selecting for KPC variants with increasing levels of resistance. Of these newer *bla*_KPC_ variants, there are four CG258 strains in our collection with *bla*_KPC-8_. Although the reason for the predominance and persistence of *bla*_KPC-2_ in our hospital system is unclear, it may be related in part to the relatively conservative use of ceftazidime and ceftazidime-avibactam compared to other beta-lactam antibiotics. The potential for a shift from a CG258-centered *K. pneumoniae* epidemic to a polyclonal epidemic was first reported in Italy in 2014, and concern has been raised in Korea (33–36).

### Analysis of *bla*_NDM-1_-carrying patient strains suggests horizontal transfer of a *bla*_NDM-1_-containing plasmid from a classic Indian *bla*_NDM-1_-positive clone into the Houston CG307 population.

The identification of the gene encoding New Delhi metallo-beta-lactamase 1 (*bla*_NDM-1_) in our patient strains is concerning because that enzyme confers very broad resistance to nearly all beta-lactam antibiotics and can be readily transferred between strains by plasmids. Since its discovery in 2009, the *bla*_NDM-1_ gene has spread globally among members of the family *Enterobacteriaceae*; however, the number of reported human infections remains relatively low. As of 6 January 2017, only 175 cases have been reported in the United States, including 2 in Texas (https://www.cdc.gov/hai/organisms/cre/TrackingCRE.html). To identify strains with *bla*_NDM-1_ in our strain sample, assembled contigs were mapped to a custom database of all published *bla*_NDM-1_-bearing plasmids. We identified 15 strains from six patients with the *bla*_NDM-1_ gene (Table 2; see Fig. S2). All contigs containing the *bla*_NDM-1_ gene are 100% identical to published plasmid sequences, indicating that no novel *bla*_NDM-1_ sequences have been introduced into or evolved *de novo* in Houston patients. Contigs from three strains were identical. Consistent with the idea that strains with the *bla*_NDM-1_ gene were imported to Houston, the majority of the *bla*_NDM-1_-containing strains belong to ST14 and ST37, two STs endemic in India, Pakistan, and elsewhere in southern Asia (55). Unexpectedly, the *bla*_NDM-1_ gene was detected in one CG307 strain. It is possible that this strain acquired the *bla*_NDM-1_ plasmid while circulating locally in Houston. Consistent with this idea, the *bla*_NDM-1_-containing plasmid in our CG307 strain (Table 2, patient F) is closely similar to a *bla*_NDM-1_-containing plasmid in two Houston patients (Table 2, patients A and B).

10.1128/mBio.00489-17.2 FIG S2  Timeline for recovery of strains with *bla*_NDM-1_-positive plasmids from six patients. The patients (A to F) are shown relative to the calendar (below). Each box represents a single episode of care. The number indicates the count of *bla*_NDM-1_ -positive strains recovered. ST14 (blue) and ST37 (red) strains are classically associated with NDM-1. CG307 strains (gray) are numerically dominant in Houston but have not been previously associated with *bla*_NDM-1_. Download FIG S2, PDF file, 0.04 MB. Copyright © 2017 Long et al. 2017 Long et al. This content is distributed under the terms of the Creative Commons Attribution 4.0 International license.

**TABLE 2:** *bla*_NDM-1_-containing strains recovered from six patients (A to F)

Strain	Patient	ST	Date collected	Source	ST geographic association(s)
528	A	ST14	12/2012	Sputum	India
537	A	ST14	12/2012	Trachea	India
553	A	SLV^a^ of ST14	12/2012	Wound	India
603	A	SLV of ST14	1/2013	Urine	India
658	A	ST14	2/2013	Blood	India
755	B	SLV of ST14	4/2013	Urine	India
764	B	ST14	4/2013	Urine	India
1010	B	ST14	10/2013	Urine	India
1273	B	SLV of ST14	1/2014	Urine	India
1145	C	ST14	12/2013	Urine	India
1402	C	SLV of ST14	4/2014	Bile	India
1426	D	ST37	5/2014	Urine	India/United Kingdom
1458	D	ST37	5/2014	Urine	India/United Kingdom
1481	E	ST906	6/2014	Urine	Israel
1844	F	ST307	10/2014	Urine	Houston

^a^ SLV, single-locus variant.

### Extensive diversity in the capsule locus.

*K. pneumoniae* capsule is an important virulence factor, and the capsule synthesis locus has considerable diversity in gene content between clonal groups (17, 49–51). Diverse associations among different *wzi* genotypes, *bla*_KPC_ genotypes, and STs have been reported, and some clonal groupings have been identified (45, 53). Recently, Holt et al. (44) proposed a new nomenclature for capsule locus genotyping (KL type) that encompasses the diversity of the known locus and described a software tool (Kaptive) for capsule locus genotyping. Using Kaptive, we identified 72 unique KL capsule locus types and potential novel variants (see Table S1A). The two most common KL capsule locus types among CG258 strains were KL106 (*n* = 259) and KL107 (*n* = 117). KL107 has the *wzi154* gene allele. Although previous studies reported that the *wzi154* allele in CG258 strains was associated with the *bla*_KPC-3_ gene, this was not the case in our strain sample. We found that almost all of our CG258 strains with a KPC gene contain *bla*_KPC-2_. The CG307 strains we studied had less diversity in the capsule locus. We found that 524 of 526 strains had the KL102 capsule locus, including all 119 ST307 strains with the *bla*_KPC-2_ gene.

In general, a given clonal group had a predominant KL capsule locus type, although some exceptions were observed (see Table S1A). CG258 is notable for having two capsule gene loci common in the group. Other clonal types with notable capsule locus diversity include ST15 (49 strains, four KL capsule locus types) and ST37 (18 strains, eight KL capsule locus types).

### *K. pneumoniae* clinical strains recovered over time from the same patient.

**FIG 5:**
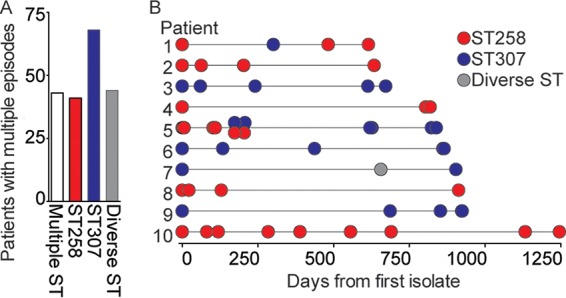
Genetic variation in ESBL-producing *K. pneumoniae* clinical strains in the same patient over time. (A) Clonal group of each strain recovered from 196 patients with multiple strains. (B) Clonal group of each isolate recovered from 10 patients (numbered 1 to 10) with multiple strains spanning the longest period. Each circle represents one isolate. The vertically overlapping circles shown for patient 5 indicate that two different strains were recovered from different specimens on the same day or consecutive days.

Many Houston Methodist patients (*n* = 357) had two or more *K. pneumoniae* strains cultured from different specimens, in some cases more than 3 years apart (Fig. 5). To investigate whether these different episodes in the same patient represent infections caused by the same or a different clone, we used the whole-genome sequence data to determine the relatedness of the organisms recovered. For this analysis, a second episode was defined as a strain isolated more than 35 days after the prior infection event. The >35-day-criterion was selected by using the Infectious Diseases Society of America definition of a late cure of cystitis (cystitis was the most common infection type in our collection) (56). Of the 196 patients with multiple strains recovered in different episodes, we found that 42 (21.4%) were infected with different clones during the study period (Fig. 5A). Similarly, 10 patients had two genomically distinct strains recovered on the same day (Fig. 5B). Consistent with the hypothesis that CG307 strains are as virulent and able to cause recurrent or chronic infections as CG258 strains, the frequency of CG307 and CG258 strains recovered from patients with multiple episodes did not differ significantly (Fisher exact test, no statistically significant difference). Of note, no simple pattern of change in capsule locus or antimicrobial resistance genes was identified among the multiple-episode patients.

### Extensive transcriptome diversity among and within clades.

**FIG 6:**
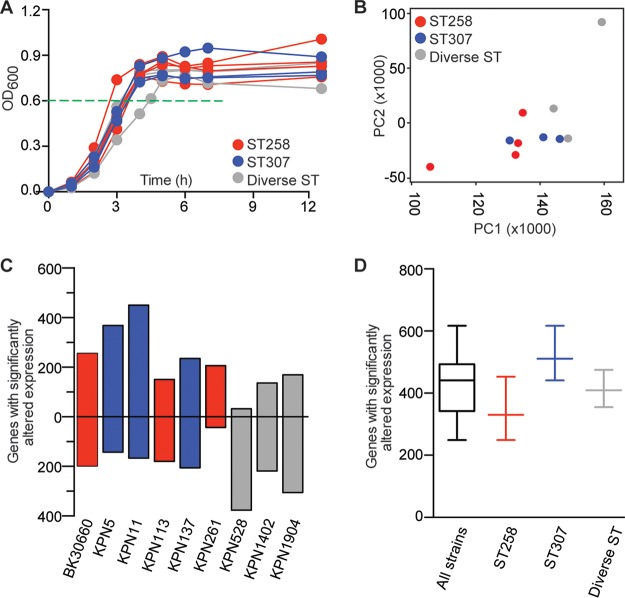
RNA-seq analysis of 10 genomically diverse ESBL-producing *K. pneumoniae* strains. Strains of CG258 (*n* = 4, red), CG307 (*n* = 3, blue), and other STs (*n* = 3, gray) were analyzed. (A) Growth curves of strains. RNA-seq analysis of duplicate cultures was done (the mean absorbance of each strain pair is shown); the growth curves of all of the strains are similar. Cells were harvested at the mid-exponential phase of growth (OD_600_ of 0.6; horizontal dashed green line). (B) RNA-seq transcriptome data for chromosomally located genes of each strain were compared to those of CG258 reference strain BK30684. Plotted are expression relationships among the strains based on principal component 1 (PC1) and principal component 2 (PC2), which account for the two largest unrelated variances in the data. The strains are distributed across both axes, showing transcriptome diversity within and between CGs. (C) Number of genes with significantly altered expression relative to reference strain BK30684. Plotted are the numbers of genes with significantly increased (positive *y* axis) and decreased (negative *y* axis) expression for each strain. (D) Box plot summarizing data from the 10 strains combined or within CGs.

Whole-genome phylogenetic analysis revealed that the ESBL-producing *K. pneumoniae* strains causing infections in our health care system could be broadly classified into three major genetic clades. To begin investigating the effect of the extensive genomic diversity on global gene expression, 10 rationally selected strains were analyzed by genome-wide transcriptome sequencing (RNA-seq) analysis. The 10 strains were selected to broadly represent the overall population structure, including the year of collection, phage content, antimicrobial resistance gene content, and numerical dominance of CG (see Table S6). Given the extensive genomic variation present, we hypothesized that each strain would have a unique transcriptome. The 10 strains had nearly superimposable growth curves, suggesting that observed differences in global transcriptome were attributable to genetic differences among strains rather than growth characteristics (Fig. 6A).

10.1128/mBio.00489-17.9 TABLE S6  Strains used for RNA-seq analysis. Genome analysis detected the presence (+) or absence (−) of the genes encoding KPC, NDM-1, and OXA-48. Download TABLE S6, PDF file, 0.1 MB. Copyright © 2017 Long et al. 2017 Long et al. This content is distributed under the terms of the Creative Commons Attribution 4.0 International license.

Transcript libraries were prepared by standard methods and sequenced to very deep coverage (mean, 22.2 [range, 14.8 to 33.6] million reads per replicate) to minimize the false discovery of significantly altered gene expression. Transcripts were counted relative to CG258 reference strain NJST258_2. Consistent with our hypothesis, principal-component analysis showed differences in the transcriptomes of these strains, both within and between the CGs (Fig. 6B). On average, each strain differentially expressed 427 genes compared to the reference strain (Fig. 6C). In the aggregate, the maximum number of differentially expressed genes was 617, indicative of the substantial diversity in these strains (Fig. 6D). Also consistent with gene content differences identified among the 10 strains, approximately half of the reads for each strain did not map to the NJST258_2 reference chromosome (mean, 46.3% reads unmapped per duplicate strain pair; range, 42.2 to 53.5%). BLAST analysis revealed that most unmapped reads derived from plasmid-borne genes, insertion sequences, and other mobile elements. Similar results were generated when reads were mapped to CG307 strain KPN11 (see Fig. S3A) or CG14 strain KPN528 (see Fig. S3B). Also, the RNA-Seq data confirmed expression of the genes encoding NDM-1, OXA-48, KPC, and yersiniabactin by strains carrying these genes (Table 2; see Fig. S3C to F and Table S6). That is, among these 10 strains, mobile gene content contributed to nearly half of all differentially expressed gene transcripts. After accounting for differences in gene content, we identified no simple patterns of transcriptome differences, either within or between the CGs.

10.1128/mBio.00489-17.3 FIG S3  RNA-seq analysis of 10 genomically diverse *K. pneumoniae* strains. (A) Box plot summarizing data from the 10 strains combined, GC258 strains, CG307 strains, and diverse ST strains. Reads were mapped to the annotated assembly of the chromosome of CG307 strain KPN11. (B) Reads were mapped to the annotated assembly of the chromosome of CG14 strain KPN528. (C, D) RNA-seq transcriptome data for reference strain BK30684 and clinical strains KPN528 (plasmid analysis detected the *bla*_NDM-1_ and OXA-48 genes) and KPN1402 (plasmid analysis detected the *bla*_NDM-1_ gene). Reads were mapped to the *bla*_NDM-1_-containing plasmid pPKPN1 or OXA-48-containing plasmid pKP112. (E) Reads were mapped to KPC-containing plasmid pNJST258C2. (F) Reads were mapped to the *ybt*-containing chromosome of CG14 strain KPN528. Normalized counts are graphed as reads per kilobase per million (RPKM) mapped reads for each gene. Download FIG S3, TIF file, 1.3 MB. Copyright © 2017 Long et al. 2017 Long et al. This content is distributed under the terms of the Creative Commons Attribution 4.0 International license.

### Mouse model of ESBL-producing *K. pneumoniae* pneumonia.

A very unexpected finding from the whole-genome sequence data was that CG307 organisms were very abundant among the ESBL-producing *K. pneumoniae* strains recovered from patients in our Houston Methodist Hospitals. To test the hypothesis that CG307 strains are as virulent as the globally disseminated and numerically common CG258 clone, reference strains KPN1411 (CG307) and KPN113 (CG258) were compared using a mouse model of pneumonia. Mice were inoculated by intranasal instillation and necropsied at 24 h, and the numbers of CFU recovered from the infection site (lung) and a site of dissemination (spleen) were determined. CFU were recovered from the lungs of all of the mice. There was no significant difference in the number of CFU cultured from the lungs of mice infected with CG307 strain KPN1411 and CG258 strain KPN113 (mean, 1.22 × 10^5^ CFU for KPN1411 compared to 8.98 × 10^4^ CFU for KPN113; data not shown). Similarly, there was no significant difference in dissemination to the spleen (four of five mice for KPN1411 compared to three of five mice for KPN113; data not shown).

**FIG 7:**
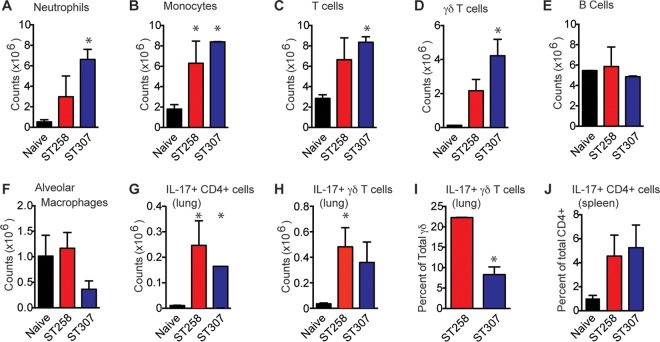
Host immune response to ESBL-producing *K. pneumoniae* in a mouse model of pneumonia. Flow cytometry analysis of leukocytes recovered from infected lungs, including neutrophils (A), monocytes (B), T cells (C), B cells (D), alveolar macrophages (E), CD4 T cells (F), γδ T cells (G), IL-17-producing CD4 T cells (H), and IL-17-producing γδ T cells (I). (J) IL-17-producing γδ T cells were also recovered from the spleen.

To further investigate host-pathogen interactions, the host immune response was examined in infected mice. Mice were inoculated by intranasal instillation, monitored for 7 days, and sacrificed, and flow cytometry analysis of immune cells recovered from their lungs and spleens was conducted (Fig. 7). Compared with uninfected control mice, animals infected with ESBL-producing *K. pneumoniae* had significantly more total CD45^+^ leukocytes in their lungs, including neutrophils (9.04-fold; Fig. 7A), monocytes (4.06-fold; Fig. 7B), T cells (2.63-fold; Fig. 7C), and γδ T cells (25.21-fold; Fig. 7D). There was no significant difference in the numbers of B lymphocytes or alveolar macrophages (Fig. 7E and F). *K. pneumoniae*-infected lungs also had significantly more γδ and CD4 T cells expressing IL-17 (9.50- and 12.07-fold, respectively, Fig. 7G and H). However, compared to the CG258 strain, the CG307 strain elicited a significantly smaller fraction of IL-17-producing γδ T cells (Fig. 7I). IL-17-expressing CD4^+^ T cells were also increased in the spleens of *K. pneumoniae*-infected animals, likely representing a reservoir of proliferating reactivated memory cells (4.78-fold; Fig. 7J). Taken together, the mouse pneumonia data demonstrate that the CG307 reference strain was as virulent as the epidemic CG258 reference strain in this disease model.

### Prediction of resistance to antimicrobial agents based on whole-genome sequence data.

**FIG 8:**
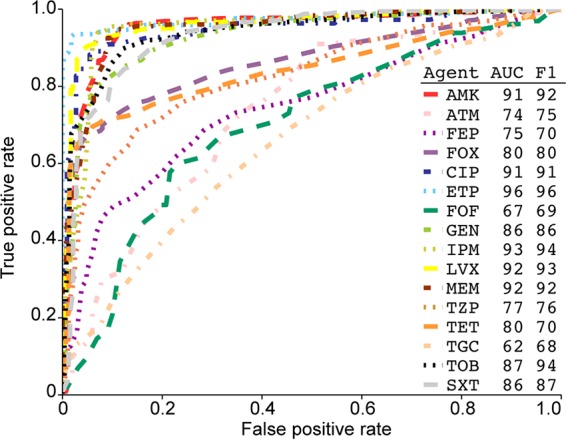
Receiver operating characteristic curves of 16 AdaBoost-based classifiers predicting antimicrobial resistance. Antibiotic agent abbreviations: AMK, amikacin; ATM, aztreonam; FEP, cefepime; FOX, cefoxitin; CIP, ciprofloxacin; ETP, ertapenem; FOF, fosfomycin; GEN, gentamicin; IPM, imipenem; LVX, levofloxacin; MEM, meropenem; TZP, piperacillin-tazobactam; TET, tetracycline; TGC, tigecycline; TOB, tobramycin; SXT, trimethoprim-sulfamethoxazole. AUC is area under the concentration-time curve. The F1 score, the harmonic mean of precision and recall, is commonly used to compare classification methods. Data are the results of a 10-fold cross validation.

The large number of genome sequences generated for this study of drug-resistant organisms gave us the opportunity to use machine learning-based classifiers to predict resistance to antimicrobial agents. To test the ability to use whole-genome sequence data to predict antimicrobial resistance phenotypes, we used the AdaBoost algorithm (57, 58) to generate classifiers for 16 agents commonly used to treat human *K. pneumoniae* infections. Results of the classifier prediction strongly correlated with the clinical phenotype, as measured by the area under the curve (AUC) and the F1 score (Fig. 8). Importantly, the prediction data generated the three highest scores for the carbapenem antibiotics.

## DISCUSSION

In the aggregate, our data demonstrate that ESBL-producing *K. pneumoniae* strains causing infections in our large hospital system over several years have substantial genomic diversity. Analysis of large, comprehensive, population-based strain samples can assist our understanding of the molecular pathogenesis of these organisms and thereby contribute to enhanced translational research.

We identified the heretofore unrecognized numerical success of CG307 strains in the Houston region (Fig. 1). CG307 strains caused more infections than the pandemic CG258 strains throughout the study period (Fig. 3). We confirmed that CG307 strains continue to be an abundant cause of infections in our hospital system by sequencing an additional 96 ESBL-producing *K. pneumoniae* strains recovered in 2017 (S. W. Long, unpublished data). Although CG307 strains have been previously identified in our area, the extent to which this clonal type has successfully coexisted with CG258 to achieve a state of codominance in our region was a very unexpected discovery. The reason(s) the CG307 clone has become abundant in Houston but not other parts of North America is not understood. CG307 strains have not been reported previously to be a prominent cause of infections in a large, unbiased population over a multiyear period. The relatively recent success of CG307 progeny in Italy and Korea has caused alarm (33, 34, 36, 47). By all measures, including the in-hospital mortality rate, the CG307 strains causing infections in Houston appear to be as virulent as CG258 strains. Over the 45-month course of our study, the incidence of ESBL-producing *K. pneumoniae* steadily increased from approximately 18 to 28 strains recovered per month (Fig. 3). There was no preferential increase in CG258 or CG307 strains compared to other ST types during the study period. This increasing incidence mirrors a trend reported elsewhere and stresses the detrimental impact of multidrug-resistant *K. pneumoniae* on human health.

The use of whole-genome sequence data to infer antimicrobial resistance phenotypes is an area of intense interest (59, 60). Two very different approaches have been pursued. The most common strategy attempts to predict phenotypes by identifying the presence or absence of genes and gene mutations conferring antimicrobial resistance (61). Although modestly successful, this strategy has two major disadvantages. First, it requires the curation of a comprehensive database that is updated as new discoveries are made. Second, this strategy is likely to fail to accurately predict resistance if the molecular mechanism is multifactorial or unknown. Since this study provides a very large, well-annotated data set, we sought an alternative approach using machine learning-based classifiers to predict antimicrobial resistance (Fig. 8). We have recently used this method to build accurate predictors of antimicrobial resistance phenotypes for several important human pathogens, including *Acinetobacter baumannii*, *Mycobacterium tuberculosis*, *Staphylococcus aureus*, and *Streptococcus pneumoniae* (62). Machine learning approaches are advantageous because they require no *a priori* knowledge of the underlying molecular mechanism of resistance and may potentially identify new antimicrobial resistance-related genomic features. The results of this study suggest that for many antibiotics, it may be possible to build diagnostically valid classifiers for the prediction of antimicrobial resistance directly from the genome sequence data. Paired with new rapid sequencing technologies, these classifiers could provide clinically actionable data (63). We also attempted to build classifiers for the prediction of other clinical phenotypes. No classifiers could be generated to accurately predict the site of infection, length of stay, chronic or repeated infection, or death. The lack of predictive classifiers for these clinical phenotypes may reflect the extensive genomic and transcriptomic diversity present in the Houston strains or the complex comorbidities present in patients with *K. pneumoniae* infections.

Our data from the mouse model of pneumonia demonstrated that the host immune response to *K. pneumoniae* is multifaceted. We found a significant infiltration of neutrophils, monocytes, and T cells in infected lungs. We also detected IL-17 production by two different T-cell subsets, CD4^+^ and γδ T cells. IL-17 is well known to play a key role in innate immunity to extracellular pathogens, particularly in patients with chronic disease or immune compromise. Chen et al. recently reported that IL-17 secretion by CD4^+^ T cells provided protection against eight strains of *K. pneumoniae* belonging to three different capsular serotypes (STs not reported) recovered from patients in Southeast Asia, an area where very few CG307 strains have been reported (64). Herein, we are the first to suggest that IL-17 production by γδ T cells may also contribute to immunity to *K. pneumoniae*. Compared to the globally pandemic CG258 strain, the locally prominent CG307 strain elicited a significantly diminished IL-17 response by γδ T cells. In addition, the lack of a yersiniabactin locus in the great majority of CG307 strains may, in part, be related to this diminished IL-17 production. Nearly all *K. pneumoniae* produce their own siderophore, enterobactin, that binds iron in the body (52). Enterobactin is susceptible to inactivation by lipocalin 2 (Lnc2), which also stimulates an inflammatory response (65). *K. pneumoniae* may acquire the yersiniabactin locus to produce a siderophore that does not bind Lnc2, providing a survival benefit in exchange for the energy required to express yersiniabactin (52, 66). By limiting IL-17 production in the immune response, CG307 *K. pneumoniae* may be enhancing the effectiveness of the enterobactin siderophore system, negating the need for yersiniabactin loci in CG307 organisms for survival and success in the host. These discoveries provide one potential explanation for the numerical success of CG307 strains among patients in Houston. That is, the CG307 clone circulating in Houston, through an undetermined genomic or transcriptomic mechanism, may elicit a different innate immune response than CG258 strains. Additional studies are under way to investigate this idea.

## MATERIALS AND METHODS

### Strain sample.

Strains were cultured from patient specimens submitted to the Diagnostic Microbiology Laboratory at Houston Methodist Hospital between September 2011 and May 2015. The Diagnostic Microbiology Laboratory serves as the central diagnostic laboratory for the 2,000-bed Houston Methodist Hospital system. Strains were collected by the research team after routine microbiology diagnostics were completed. All of the strains collected were ESBL-producing *K. pneumoniae*, as determined by automated methods (Phoenix ESBL test; BD). A summary of the isolate collection dates and sources is provided in Table S1A. Strains were collected and patient data were examined under human subjects protocol IRB1010-0199 as approved by the Institutional Review Board, Houston Methodist Research Institute.

### DNA extraction and whole-genome sequencing.

Genomic and plasmid DNA was extracted in accordance with the Gram-negative protocol of the DNeasy Blood and Tissue kit (Qiagen). DNA was quantitated with the Qubit (Invitrogen) instrument, and whole-genome sequencing libraries were prepared with NexteraXT reagents (Illumina). Libraries were sequenced with Illumina MiSeq and NextSeq 500 instruments.

### Bioinformatic analysis.

Initial bioinformatic analysis was performed on the high-performance computer cluster in the Houston Methodist Research Institute, a Beowulf cluster with 336 CPU cores and 1.8 TB of RAM. FASTQ files were processed with Trimmomatic (v 0.35) to remove adapter sequence contamination and Musket (v 1.1) to correct short-read sequence errors (67, 68). SMALT (v 0.7.6) was used to align reads, and FreeBayes (v 0.9.20) was used to identify variants (69). The in-house-developed scripts prephix and phrecon were used to generate multi-FASTA files of SNPs for phylogenetic tree generation (https://github.com/codinghedgehog/). Phylogenetic trees were generated with FastTreeMP (v 2.1.8) using double precision and annotated with CLC Genomics workbench 9.5.2 (Qiagen) (70). Short-read sequence typing for bacterial pathogens (SRST2) (71) was used to identify the MLST assignment of each strain with the *K. pneumoniae* database of 2,470 STs available on 4 November 2016 curated at the Institut Pasteur (http://bigsdb.web.pasteur.fr/klebsiella/). SRST2 was also used to identify antibiotic resistance gene content and plasmid replicon content by using the SRST2 gene database files ARGannot.r1.fasta and PlasmidFinder.fasta. Reads were assembled into contigs with SPAdes v.3.6.2 (72). Kaptive was used to identify the capsule locus genotype and capsule locus gene content from assembled contigs (50). Kleborate was used to identify yersiniabactin, colibactin, and other siderophore locus content from assembled contigs (52). MEGA7 and MEGA-CC were used to generate distance matrices for pairwise distance analysis (73, 74). Gene content analysis was performed with the Proteome Comparison tool in PATRIC (75).

### Pacific Biosciences genome sequence data.

Strains were sequenced to closure by the Pacific Biosciences SMRT long-read strategy in the laboratory of C. Mason at Weill Cornell Medical College. This technology permitted closed assembly of the core chromosome and plasmid DNA sequences from the target strains. Polished contigs were generated with HGAP. The plasmid sequences were compared to the NT database at NCBI with BLAST.

### Genome-wide RNA transcript analysis.

RNA-Seq analysis was conducted as previously described (76). Briefly, 10 strains were selected to be broadly representative of our population, on the basis of the year of collection, phage type, CRE type, *bla*_NDM-1_ type, and numerical dominance of CGs (see Table S6). Two of these 10 strains were previously sequenced to closure (17), and 1 was sequenced to closure in this study with PacBio as described above. Duplicate cultures of each strain were inoculated into prewarmed LB medium and grown at 37°C to the mid-exponential phase (optical density at 600 nm [OD_600_] of 0.60 to 0.65). Total RNA was extracted with an RNeasy kit (Qiagen) according to the manufacturer’s instructions, and RNA-seq libraries were prepared with an Epicenter ScriptSeq kit (Illumina). The libraries were sequenced by using a 75-bp single-end protocol with a high-output v 3 reagent kit (Illumina). To generate a sufficient number of sequence reads for transcript analysis (average of 12 million reads per library), two runs were performed with one MiSeq instrument at the default software settings for the Generate FASTQ on instrument workflow. The two FASTQ files generated for each library were concatenated, and the number and quality of sequence reads were determined with FastQC (Babraham Bioscience Technologies, Cambridge, United Kingdom). The RNA transcripts were quantified from the concatenated FASTQ files with the default software settings relative to the chromosome of strain BK30684 with CLC Genomics Workbench v 9.5.2 (Qiagen). Differences of >1.5-fold by Baggerly’s test and a *P* value of <0.05 after applying Bonferroni’s correction for multiple comparisons were considered significant. Strain BK30684 was selected as the chromosome reference because the genome has been sequenced to closure, it is genetically representative of the globally disseminated CG258 clone, and it has been used in previous pathogenomic studies (17). To confirm that the many differences in gene expression observed between strains were due to differences in gene content, RNA-Seq reads were also mapped to CG307 reference strain KPN11 and CG14 reference strain KPN528 (see Table S3). To confirm expression of the KPC gene, reads were mapped to reference plasmid pNFST258C2 (GenBank accession no. CP006919.1). To confirm expression of the *bla*_NDM-1_ and OXA48 genes, reads were mapped to reference plasmids pPKPN1 (GenBank accession no. CP006799.1) and pKP112 (GenBank accession no. LN864819.1).

### Mouse model of pneumonia.

A mouse model of pneumonia was used to compare the virulence of reference CG307 and CG258 strains KPN1411 and KPN113, respectively. C57BL/6 mice were inoculated by intranasal instillation of 1 × 10^8^ CFU in 30 µl (15 µl in each naris). At 24 h postinoculation, mice were sacrificed. The lungs and spleens were removed, homogenized (Omni), serially diluted in sterile phosphate-buffered saline, plated on tryptic soy agar supplemented with 5% sheep blood, and incubated overnight at 37 C. CFU were counted, graphed as the mean ± the standard error of the mean, and statistically compared by using the Mann-Whitney test (GraphPad). Similarly, mice were infected and sacrificed after 7 days and their lungs were harvested for flow cytometry analysis by standard methods (77).

### Preparation of genomic data for antimicrobial resistance prediction.

Antimicrobial susceptibility testing for each strain was performed as part of the routine clinical workup. Machine learning-based classifiers for antimicrobial resistance phenotype prediction were created as previously described (62), by using the genomic data and clinical antimicrobial susceptibility data generated in this study. Briefly, the 1,777 genomes were assembled by using the PATRIC Full SPAdes assembly strategy and annotated by using the PATRIC annotation service (75). For each antimicrobial agent, the genomes were evenly divided into resistant and susceptible sets, matrices were built with 15-bp k-mers, and 10 iterative rounds of the AdaBoost algorithm were performed. Tenfold cross validation was applied to the data set for each classifier (62). The final set of classifiers generated for each antimicrobial agent is accessible in the PATRIC and RAST annotation services.

### Accession number(s).

The whole genome sequence FASTQ files, RNAseq FASTQ files, and genomes of the strains sequenced by PacBio (see Table S3) have been deposited in the NCBI database under BioProject no. PRJNA376414.
