# Supplementary figures and images for: Population Genomic Analysis of 1,777 Extended-Spectrum Beta-Lactamase-Producing Klebsiella pneumoniae Isolates, Houston, Texas: Unexpected Abundance of Clonal Group 307
Source: mBio. 2017 May 16;8(3):e00489-17. doi: 10.1128/mBio.00489-17 (PMC5433097; doi:10.1128/mBio.00489-17)

### Figure S1

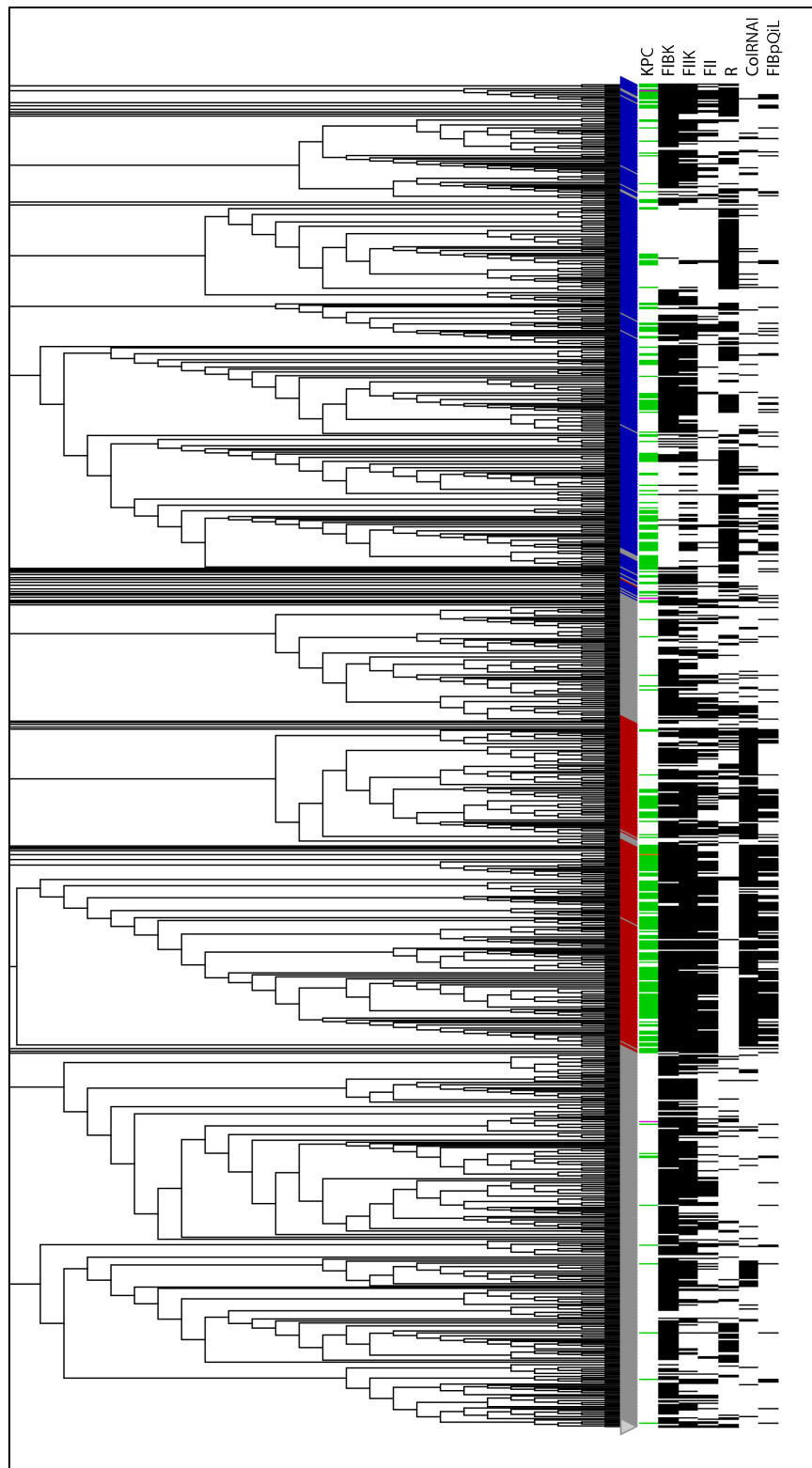

Supplement: FIG S1 [file mbo003173305sf1.pdf]

Figure S2

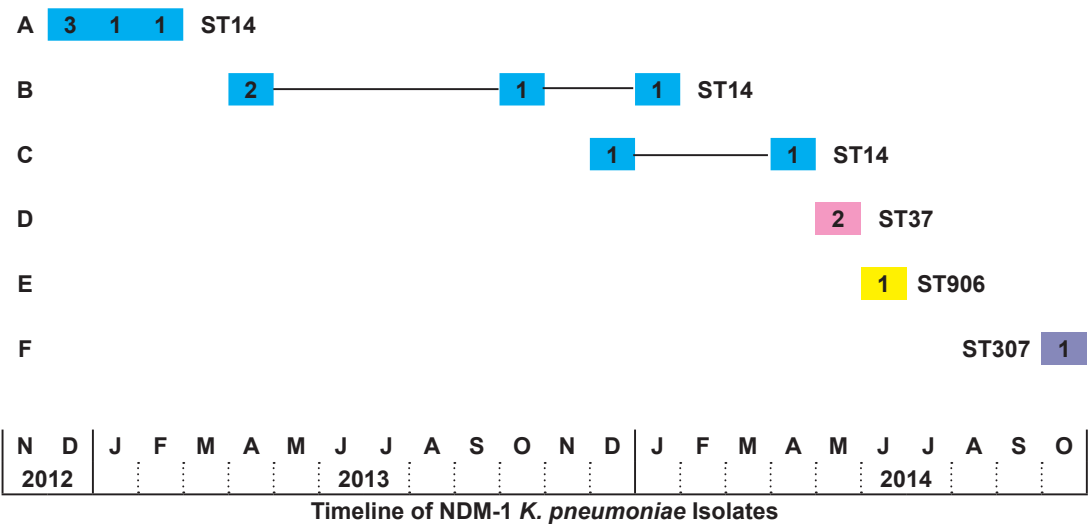

Supplement: FIG S2 [file mbo003173305sf2.pdf]

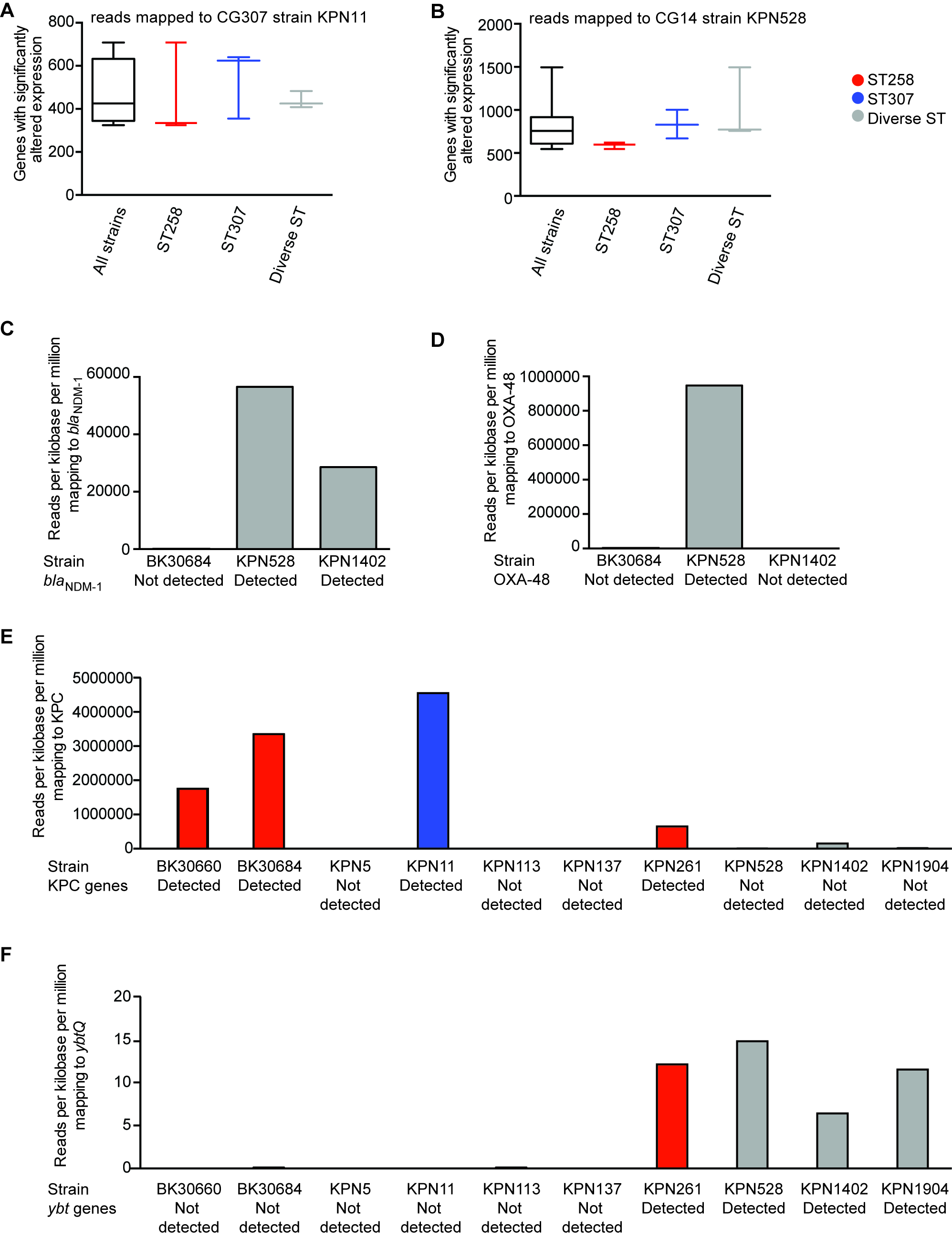

Supplement: FIG S3 [file mbo003173305sf3.tif]
