# Supplementary material for: Population Genomic Analysis of 1,777 Extended-Spectrum Beta-Lactamase-Producing Klebsiella pneumoniae Isolates, Houston, Texas: Unexpected Abundance of Clonal Group 307
Source: mBio. 2017 May 16;8(3):e00489-17. doi: 10.1128/mBio.00489-17 (PMC5433097; doi:10.1128/mBio.00489-17)
Supplement: TABLE S2 [file mbo003173305st2.pdf]

**Table S2. Sequence types of strains recovered in this study.**

| ST | Strains (n) |
|---|---|
| 11 | 1 |
| 13 | 25 |
| 14 | 13 |
| 15 | 49 |
| 16 | 92 |
| 17 | 17 |
| 20 | 6 |
| 22 | 6 |
| 25 | 12 |
| 26 | 1 |
| 29 | 5 |
| 35 | 5 |
| 36 | 10 |
| 37 | 18 |
| 39 | 1 |
| 42 | 5 |
| 45 | 16 |
| 48 | 1 |
| 76 | 2 |

| | |
|---|---|
| 101 | 3 |
| 111 | 6 |
| 134 | 3 |
| 138 | 1 |
| 147 | 20 |
| 151 | 1 |
| 187 | 2 |
| 196 | 1 |
| 219 | 1 |
| 234 | 3 |
| 258 | 382 |
| 261 | 1 |
| 273 | 5 |
| 280 | 32 |
| 295 | 2 |
| 307 | 526 |
| 321 | 1 |
| 323 | 3 |
| 327 | 6 |
| 336 | 1 |
| 340 | 1 |

| | |
|---|---|
| 342 | 1 |
| 348 | 1 |
| 392 | 12 |
| 405 | 9 |
| 412 | 1 |
| 418 | 1 |
| 429 | 2 |
| 432 | 2 |
| 454 | 2 |
| 461 | 1 |
| 462 | 7 |
| 471 | 1 |
| 476 | 2 |
| 551 | 9 |
| 611 | 1 |
| 628 | 9 |
| 656 | 1 |
| 661 | 1 |
| 681 | 2 |
| 716 | 5 |
| 753 | 1 |

| | |
|---|---|
| 784 | 2 |
| 788 | 1 |
| 791 | 1 |
| 869 | 2 |
| 874 | 1 |
| 880 | 1 |
| 884 | 4 |
| 895 | 2 |
| 896 | 3 |
| 906 | 1 |
| 978 | 2 |
| 985 | 1 |
| 1017 | 3 |
| 1107 | 1 |
| 1114 | 1 |
| 1174 | 1 |
| 1213 | 2 |
| 1228 | 1 |
| 1296 | 1 |
| 1412 | 4 |
| 1427 | 7 |

| | |
|---|---|
| 1440 | 8 |
| 1444 | 1 |
| 1456 | 2 |
| 1486 | 1 |
| 1500 | 1 |
| 1602 | 1 |
| 1694 | 2 |
| 1758 | 2 |
| 1777 | 1 |
| 1808 | 1 |
| 1823 | 1 |
| 1887 | 3 |
| 1962 | 1 |
| 2133 | 1 |
| 2185 | 1 |
| 2202 | 2 |
| 2351 | 2 |
| 1234* | 7 |
| 147* | 1 |
| 16* | 1 |
| 1662* | 1 |

| | |
|---|---|
| 17* | 1 |
| 2079* | 1 |
| 2289* | 1 |
| 258* | 2 |
| 307* | 18 |
| 36* | 1 |
| 395* | 1 |
| 414* | 1 |
| 42* | 3 |
| 45* | 1 |
| 461* | 2 |
| 551* | 1 |
| 560* | 1 |
| 896* | 1 |
| NF | 51 |
| NF* | 251 |

Abbreviations: NF, novel allele combination. * indicates polymorphism in allele sequence.
