# Supplementary material for: Population Genomic Analysis of 1,777 Extended-Spectrum Beta-Lactamase-Producing Klebsiella pneumoniae Isolates, Houston, Texas: Unexpected Abundance of Clonal Group 307
Source: mBio. 2017 May 16;8(3):e00489-17. doi: 10.1128/mBio.00489-17 (PMC5433097; doi:10.1128/mBio.00489-17)
Supplement: TABLE S3 [file mbo003173305st3.pdf]

Table S3. Strains sequenced to closure.

Strain	ST	Chr/Plasmid	Contig Size(s) (bp)
KPN11	307	Chr	5,069,972<br>270,681
		pKPN11-1	330,476
		pKPN11-2	79,539
KPN528	14	Chr	5,383,018
		pKPN528-1	292,471
		pKPN528-2	221,428
		pKPN528-3	76,158
KPN1481	906	Chr	5,554,150
		pKPN1481-1	347,748
		pKPN1481-2	161,100
		pKPN1481-3	20,380
		pKPN1481-4	12,376
		pKPN1481-5	10,373
KPN1482	37	Chr	5,591,645
		pKPN1482-1	180,210
		pKPN1482-2	97,202
		pKPN1482-3	74,177
		pKPN1482-4	12,540
		pKPN1482-5	9,510
BK13043	258	Chr	5,594,550

		**pBK13043-1**	**232,540**
		**pBK13043-2**	**57,580**
		**pBK13043-3**	**11,984**

Abbreviations: ST, multilocus sequence type; Chr, chromosome.
