# Supplementary material for: Population Genomic Analysis of 1,777 Extended-Spectrum Beta-Lactamase-Producing Klebsiella pneumoniae Isolates, Houston, Texas: Unexpected Abundance of Clonal Group 307
Source: mBio. 2017 May 16;8(3):e00489-17. doi: 10.1128/mBio.00489-17 (PMC5433097; doi:10.1128/mBio.00489-17)
Supplement: TABLE S4 [file mbo003173305st4.pdf]

Table S4. Annotated genes present in NJST258_2 but not CG307 strain KPN11.

ref_genome_patric_id	ref_genomeFunction annotated in PATRIC	start	end
fig|1420012.6.peg.636	Mobile element protein	654753	655952
fig|1420012.6.peg.637	orf, conserved hypothetical protein	655962	656150
fig|1420012.6.peg.769	Mobile element protein	785412	785564
fig|1420012.6.peg.770	Chromosome partitioning ATPase in PFGI-1-like cluster, ParA-like	786234	787106
fig|1420012.6.peg.773	Protein with ParB-like nuclease domain in PFGI-1-like cluster	788846	790468
fig|1420012.6.peg.775	FIG141751: hypothetical protein in PFGI-1-like cluster	791052	792269
fig|1420012.6.peg.781	FIG141694: hypothetical protein in PFGI-1-like cluster	798306	799028
fig|1420012.6.peg.782	Putative uncharacterized protein STY4534 (Putative uncharacterized protein)	799745	800197
fig|1420012.6.peg.783	PilL	800321	800890
fig|1420012.6.peg.785	probable exported protein STY4558	801744	802361
fig|1420012.6.peg.788	Coupling protein VirD4, ATPase required for T-DNA transfer	803456	805555
fig|1420012.6.peg.790	putative membrane protein	805815	806573
fig|1420012.6.peg.797	putative exported protein	809830	810483
fig|1420012.6.peg.799	putative exported protein	811328	812809
fig|1420012.6.peg.800	putative lipoprotein	812823	813221
fig|1420012.6.peg.801	Type IV secretory pathway, VirB4 components	813221	815995
fig|1420012.6.peg.805	Glycosaminoglycan attachment site	817441	818808
fig|1420012.6.peg.810	corresponds to STY4575 from Accession AL513382: Salmonella typhi CT18	823995	824390
fig|1420012.6.peg.814	putative membrane protein	827106	828635
fig|1420012.6.peg.820	COG1396: Predicted transcriptional regulators	830907	831101
fig|1420012.6.peg.828	Antirestriction protein	837438	838418
fig|1420012.6.peg.830	Mobile element protein	839859	840806
fig|1420012.6.peg.1747	O antigen biosynthesis rhamnosyltransferase rfbN (EC 2.4.1.-)	1804929	1805837
fig|1420012.6.peg.1749	predicted glycosyltransferase	1806896	1807657
fig|1420012.6.peg.1754	dTDP-4-dehydrorhamnose reductase (EC 1.1.1.133)	1812250	1813140
fig|1420012.6.peg.1755	dTDP-4-dehydrorhamnose 3,5-epimerase (EC 5.1.3.13)	1813155	1813709
fig|1420012.6.peg.1763	capsular polysaccharide biosynthesis protein	1820697	1821731
fig|1420012.6.peg.1764	Phage tail fibers	1822000	1823577
fig|1420012.6.peg.1832	Conjugal transfer protein traA	1887562	1889082
fig|1420012.6.peg.1837	IncI1 plasmid conjugative transfer prepilin PilS	1893277	1893846

fig|1420012.6.peg.1838	PilV-like protein	1894050	1895609
fig|1420012.6.peg.1839	Type III restriction-modification system restriction subunit (EC 3.1.21.5)	1896072	1899032
fig|1420012.6.peg.1840	Type III restriction-modification system methylation subunit (EC 2.1.1.72)	1899042	1900892
fig|1420012.6.peg.1841	SpnT	1901086	1902474
fig|1420012.6.peg.2030	Uropathogenic specific protein	2090595	2091950
fig|1420012.6.peg.2035	Uropathogenic specific protein	2094003	2095283
fig|1420012.6.peg.2043	serine/threonine kinase	2099278	2100171
fig|1420012.6.peg.2045	serine/threonine kinase	2100355	2101248
fig|1420012.6.peg.2046	serine/threonine kinase	2101424	2102317
fig|1420012.6.peg.2047	serine/threonine kinase	2102493	2103383
fig|1420012.6.peg.2542	Phosphoglycerate mutase (EC 5.4.2.1)	2591361	2591792
fig|1420012.6.peg.2544	Probable signal peptide protein	2593420	2594181
fig|1420012.6.peg.2620	Acetyltransferase, GNAT family	2668462	2668875
fig|1420012.6.peg.2720	expressed protein	2760682	2761848
fig|1420012.6.peg.3029	Cold shock protein CspG	3054694	3054882
fig|1420012.6.peg.3031	Beta-mannosidase (EC 3.2.1.25)	3055267	3055404
fig|1420012.6.peg.3101	D-glycerate 2-kinase (EC 2.7.1.165)	3124051	3125310
fig|1420012.6.peg.3485	Prophage P2 OGR protein	3520689	3520907
fig|1420012.6.peg.3725	orf, conserved hypothetical protein	3761888	3762076
fig|1420012.6.peg.3726	Mobile element protein	3762086	3763285
fig|1420012.6.peg.3900	Response regulator receiver protein	3933204	3933821
fig|1420012.6.peg.3906	Transcriptional regulator, LuxR family	3940054	3940689
fig|1420012.6.peg.4167	DNA polymerase IV	4207777	4207947
fig|1420012.6.peg.4466	SSU ribosomal protein S20p	4532925	4533110
fig|1420012.6.peg.4541	Regulator of L-galactonate catabolism YjjM	4610418	4611332
fig|1420012.6.peg.4629	ORF25	4701142	4701780
