# Supplementary material for: Population Genomic Analysis of 1,777 Extended-Spectrum Beta-Lactamase-Producing Klebsiella pneumoniae Isolates, Houston, Texas: Unexpected Abundance of Clonal Group 307
Source: mBio. 2017 May 16;8(3):e00489-17. doi: 10.1128/mBio.00489-17 (PMC5433097; doi:10.1128/mBio.00489-17)
Supplement: TABLE S5 [file mbo003173305st5.pdf]

Table S5. Annotated genes present in KPN11 but not in NJST258_2.

ref_genome_patric_id	ref_genomeFunction annotated in PATRIC	start	End
fig|1420013.7.peg.13	Putative cytoplasmic protein	7519	8499
fig|1420013.7.peg.14	Holliday junction resolvase / Crossover junction endodeoxyribonuclease rusA (EC 3.1.22.-)	8512	8889
fig|1420013.7.peg.16	Replication protein O	9705	10658
fig|1420013.7.peg.17	Orf33	11065	11526
fig|1420013.7.peg.19	regulatory protein	11901	12596
fig|1420013.7.peg.34	VgrG protein	24884	25348
fig|1420013.7.peg.60	ClpB protein	50454	50576
fig|1420013.7.peg.225	Phosphoribosylformylglycinamidine synthase	230240	230389
fig|1420013.7.peg.245	COG2116: Formate/nitrite family of transporters	253932	254312
fig|1420013.7.peg.263	62kDa structural protein	267609	269033
fig|1420013.7.peg.277	endodeoxyribonuclease RusA	275420	275776
fig|1420013.7.peg.291	putative DNA-binding protein	281322	281657
fig|1420013.7.peg.293	Primosomal protein I	282417	283325
fig|1420013.7.peg.301	Exodeoxyribonuclease VIII (EC 3.1.11.-)	286941	289991
fig|1420013.7.peg.302	Gifsy-2 prophage protein	290004	291092
fig|1420013.7.peg.467	Formate hydrogenlyase transcriptional activator	455786	456046
fig|1420013.7.peg.494	DNA mismatch repair protein MutS	484846	484935
fig|1420013.7.peg.597	Uncharacterized protein ImpA	596880	597299
fig|1420013.7.peg.678	Diaminopimelate decarboxylase (EC 4.1.1.20)	676382	676534
fig|1420013.7.peg.791	Outer membrane porin, OprD family	785507	785632
fig|1420013.7.peg.1339	Ankyrin	1331096	1331206
fig|1420013.7.peg.1380	Membrane fusion component of tripartite multidrug resistance system	1376993	1377130
fig|1420013.7.peg.1457	PTS system, maltose and glucose-specific IIC component	1453895	1454035
fig|1420013.7.peg.1535	DNA-damage-inducible protein D	1531123	1531359
fig|1420013.7.peg.1717	Amidohydrolase 2	1725952	1726950
fig|1420013.7.peg.1765	Ubiquinone biosynthesis monooxygenase UbiB	1781219	1781608
fig|1420013.7.peg.1905	Outer membrane usher protein fimD precursor	1937528	1937989
fig|1420013.7.peg.1940	Melibiose carrier protein, Na+/melibiose symporter	1972593	1972856

fig|1420013.7.peg.2133	Type I restriction-modification system, DNA-methyltransferase subunit M (EC 2.1.1.72)	2171563	2173062
fig|1420013.7.peg.2134	Type I restriction-modification system, DNA-methyltransferase subunit M (EC 2.1.1.72)	2173068	2174558
fig|1420013.7.peg.2135	Type I restriction-modification system, specificity subunit S (EC 3.1.21.3)	2174559	2175761
fig|1420013.7.peg.2136	Type I restriction-modification system, restriction subunit R (EC 3.1.21.3)	2175761	2178823
fig|1420013.7.peg.2151	probable membrane protein YPO2297	2188809	2191118
fig|1420013.7.peg.2152	probable ATP /GTP binding protein	2191122	2192072
fig|1420013.7.peg.2153	probable ATP /GTP binding protein	2192288	2192437
fig|1420013.7.peg.2158	NgrB	2202059	2202697
fig|1420013.7.peg.2159	NgrB	2202694	2202933
fig|1420013.7.peg.2160	predicted DNA-binding transcriptional regulator	2203177	2203893
fig|1420013.7.peg.2166	YfjZ protein (antitoxin to YpjF)	2206904	2207245
fig|1420013.7.peg.2167	YpjF toxin protein	2207266	2207607
fig|1420013.7.peg.2174	Xylose isomerase (EC 5.3.1.5)	2216142	2216456
fig|1420013.7.peg.2224	GntR family transcriptional regulator YjiR @ Transcriptional regulator, GntR family domain	2268293	2268700
fig|1420013.7.peg.2258	Putative oxidoreductase in 4-hydroxyproline catabolic gene cluster	2299662	2300924
fig|1420013.7.peg.2259	Hypothetical, similar to sarcosine oxidase alpha subunit, 2Fe-2S domain	2300905	2301168
fig|1420013.7.peg.2260	D-amino-acid oxidase (EC 1.4.3.3)	2301165	2302274
fig|1420013.7.peg.2261	4-hydroxyproline epimerase (EC 5.1.1.8)	2302271	2303218
fig|1420013.7.peg.2592	Alpha-D-GlcNAc alpha-1,2-L-rhamnosyltransferase (EC 2.4.1.-)	2660848	2661948
fig|1420013.7.peg.2594	Putative glycosyltransferase protein	2663140	2664165
fig|1420013.7.peg.2596	O-antigen flippase Wzx	2665531	2666748
fig|1420013.7.peg.2598	polysaccharide export protein	2668843	2669397
fig|1420013.7.peg.2599	Capsular polysaccharide synthesis enzyme CpsB	2669394	2670680
fig|1420013.7.peg.2752	4-hydroxybenzoate transporter	2818431	2818709
fig|1420013.7.peg.2753	4-hydroxybenzoate transporter	2818715	2819050
fig|1420013.7.peg.2757	3-oxosteroid 1-dehydrogenase (EC 1.3.99.4)	2821977	2822099
fig|1420013.7.peg.2804	Maltose/maltodextrin ABC transporter, permease protein MalG	2868718	2868843
fig|1420013.7.peg.2864	putative DNA methylase	2930202	2930729
fig|1420013.7.peg.2869	TolA protein	2932711	2933682

fig|1420013.7.peg.2902	Sb8	2952878	2953195
fig|1420013.7.peg.2913	COG2116: Formate/nitrite family of transporters	2960562	2960942
fig|1420013.7.peg.2977	putative membrane protein	3028855	3029142
fig|1420013.7.peg.2978	Helix-turn-helix motif	3029143	3029505
fig|1420013.7.peg.3007	beta-lactamase domain protein	3060820	3061920
fig|1420013.7.peg.3009	Putative inner membrane protein	3062310	3062558
fig|1420013.7.peg.3016	Permease of the drug/metabolite transporter (DMT) superfamily	3072375	3073298
fig|1420013.7.peg.3060	Isochorismate synthase (EC 5.4.4.2) [enterobactin] siderophore @ Isochorismate synthase (EC 5.4.4.2) of siderophore biosynthesis	3123974	3124363
fig|1420013.7.peg.3196	TolA protein	3265787	3267112
fig|1420013.7.peg.3219	Putative molybdenum transport ATP-binding protein modF	3290803	3290958
fig|1420013.7.peg.3285	Transketolase, C-terminal section (EC 2.2.1.1)	3356732	3356890
fig|1420013.7.peg.3314	Transcriptional regulator, LacI family	3387175	3387417
fig|1420013.7.peg.3437	Alkanesulfonates-binding protein	3528879	3529010
fig|1420013.7.peg.3460	T1SS secreted agglutinin RTX	3554015	3566473
fig|1420013.7.peg.3463	Type I secretion system, membrane fusion protein LapC	3570219	3571439
fig|1420013.7.peg.3464	poly (glycerol-phosphate) alpha-glucosyltransferase	3571493	3574627
fig|1420013.7.peg.3520	COG1309: Transcriptional regulator	3629350	3629796
fig|1420013.7.peg.3521	Arginase (EC 3.5.3.1)	3630017	3630880
fig|1420013.7.peg.3591	Candidate type III effector Hop protein	3699412	3701130
fig|1420013.7.peg.3592	2-keto-3-deoxygluconate permease (KDG permease)	3701223	3702242
fig|1420013.7.peg.3594	Ribose 5-phosphate isomerase B (EC 5.3.1.6)	3702647	3703105
fig|1420013.7.peg.3596	Transketolase (EC 2.2.1.1)	3704106	3704252
fig|1420013.7.peg.4017	Small heat shock protein	4123502	4123882
fig|1420013.7.peg.4143	Xylulose kinase (EC 2.7.1.17)	4242382	4242762
fig|1420013.7.peg.4218	Multidrug resistance protein ErmB	4314002	4314142
fig|1420013.7.peg.4236	transcriptional regulator, LacI family	4330648	4330947
fig|1420013.7.peg.4329	Muconate cycloisomerase (EC 5.5.1.1)	4421689	4421907
fig|1420013.7.peg.4336	Respiratory nitrate reductase alpha chain (EC 1.7.99.4)	4430622	4431461
fig|1420013.7.peg.4341	N-hydroxyarylamine O-acetyltransferase (EC 2.3.1.118)	4435522	4435770
fig|1420013.7.peg.4402	Benzoate transport protein	4490473	4490706
fig|1420013.7.peg.4472	Acid shock protein precursor	4555436	4555729

fig|1420013.7.peg.4483	Alcohol dehydrogenase (EC 1.1.1.1)	4563022	4563144
fig|1420013.7.peg.4497	Permeases of the major facilitator superfamily	4574569	4574790
fig|1420013.7.peg.4532	probable integral membrane protein NMA1777	4611234	4612118
fig|1420013.7.peg.4556	ABC transporter related	4632972	4633064
fig|1420013.7.peg.4740	Outer membrane protein	4827634	4828068
fig|1420013.7.peg.4982	Lipopolysaccharide biosynthesis protein WzxC	5066006	5067310
fig|1420013.7.peg.4989	Low molecular weight protein-tyrosine-phosphatase Wzb (EC 3.1.3.48)	4878	5009
fig|1420013.7.peg.5002	periplasmic sensor signal transduction histidine kinase	19522	19962
fig|1420013.7.peg.5010	Multidrug transporter MdtB	29094	29405
fig|1420013.7.peg.5067	tRNA-dihydrouridine synthase C (EC 1.-.-.-)	93272	93553
