# Supplementary material for: Population Genomic Analysis of 1,777 Extended-Spectrum Beta-Lactamase-Producing Klebsiella pneumoniae Isolates, Houston, Texas: Unexpected Abundance of Clonal Group 307
Source: mBio. 2017 May 16;8(3):e00489-17. doi: 10.1128/mBio.00489-17 (PMC5433097; doi:10.1128/mBio.00489-17)
Supplement: TABLE S6 [file mbo003173305st6.pdf]

Table S6. Strains used for RNA sequence analysis. Genome analysis detected the presence (+) or absence (-) of the genes encoding the NDM-1 and OXA-49 beta-lactamases.

Strain	Clonal group	State	Year	NDM-1	OXA-48
BK30660	258	NJ	2010	-	-
BK30684	258	NJ	2010	-	-
KPN5	307	TX	2011	-	-
KPN11	307	TX	2011	-	-
KPN113	258	TX	2011	-	-
KPN137	307	TX	2011	-	-
KPN261	258	TX	2012	-	-
KPN528	14	TX	2012	+	+
KPN1402	14	TX	2014	+	-
KPN1904	327	TX	2014	-	-
